# An Insight into the Proteome of Uveal Melanoma-Derived Ectosomes Reveals the Presence of Potentially Useful Biomarkers

**DOI:** 10.3390/ijms20153789

**Published:** 2019-08-02

**Authors:** Magdalena Surman, Dorota Hoja-Łukowicz, Sabina Szwed, Sylwia Kędracka-Krok, Urszula Jankowska, Magdalena Kurtyka, Anna Drożdż, Anna Lityńska, Ewa Stępień, Małgorzata Przybyło

**Affiliations:** 1 Department of Glycoconjugate Biochemistry, Institute of Zoology and Biomedical Research, Faculty of Biology, Jagiellonian University, 30-387 Kraków, Poland; 2 Department of Physical Biochemistry, Faculty of Biochemistry, Biophysics and Biotechnology, Jagiellonian University, 30-387 Kraków, Poland; 3 Laboratory of Proteomics and Mass Spectrometry, Malopolska Centre of Biotechnology, Jagiellonian University, 30-387 Kraków, Poland; 4 Department of Medical Physics, M. Smoluchowski Institute of Physics, Faculty of Physics, Astronomy and Applied Computer Science, Jagiellonian University, 30-348 Kraków, Poland

**Keywords:** uveal melanoma, ectosomes, proteomics, glycosylation, extracellular vesicles

## Abstract

Cancer cells are known to release extracellular vesicles that often promote disease development and progression. The present study investigated the protein content and glycosylation pattern of ectosomes released in vitro by a human primary uveal melanoma Mel202 cell line. Ectosomes released by Mel202 cells were isolated from conditioned media using sequential centrifugation, and a nano-LC-MS/MS approach was used to determine their protein content. Subsequently, proteins from ectosomes, the whole cell extracts, and the membrane fractions were probed with a panel of lectins using Western blotting and flow cytometry to reveal characteristic glycan structures. As many as 2527 unique proteins were identified, and many of them are known to be involved in cancer cell proliferation and altered metabolism, tumor invasion, metastasis, or drug resistance. Lectin-based studies revealed a distinct glycosylation pattern between Mel202-derived ectosomes and the parental cell membranes. Selective enrichment of ectosomal proteins with bisected complex type N-glycans and α2,6-linked sialic acids may be significant for ectosome formation and sequestration. Differences in the surface glycosylation of Mel202 cells and ectosomes supports recent findings that the budding of ectosomes occurs within strictly determined fragments of the plasma membrane, and thus ectosomes contain a unique protein and glycan composition.

## 1. Introduction

Ocular melanomas are rare but potentially life-threatening malignancies that arise from melanocytes of the uveal tract, conjunctiva, or orbit. Uveal melanoma (UM) is the most common type of ocular melanoma that accounts for 85–95% of ocular melanoma cases, but only for 3–5% of all melanomas. Despite the use of radiation or surgical therapy, up to 50% of patients with primary UM will develop metastatic disease [1], for which there is no standard therapy and no treatment that has been shown to improve overall survival. Therefore, a growing amount of research concerns novel targeted therapies and prognostic indicators of UM metastatic spread. Currently used demographic, clinical, and histopathological features of UM present insufficient predictive value; however, when combined with cytogenetic analysis, they may allow for identifying UM patients with the increased risk of metastasis [2].

Advances in UM genomics and proteomics have generated many candidate biomarkers that are measurable in different body fluids of UM patients. Although only a few proteomic studies have been carried out, they identified hundreds of proteins involved in UM cell proliferation, adhesion, and metastasis [3,4,5,6]. Most studies have been performed using cell lines [3,7,8,9,10], with only three studies using UM tissue [6,11,12]. However, novel proteomic technologies provide the opportunity for high-throughput quantitative studies to be performed using various biological material.

A variety of bioactive molecules involved in carcinogenesis, as well as potential disease biomarkers, can be transferred via extracellular vesicles (EVs), which are small, membrane-enclosed particles that are released from almost all cell types. Tumor-derived EVs play functional roles in neoplastic transformation, tumor invasion and metastasis, angiogenesis, escape from immune surveillance, and drug resistance [13,14]. Following their in vivo release to the intercellular space, EVs can be detected in body fluids or isolated from conditioned media of cultured cells, and such accessibility contributes to their prognostic and diagnostic value for particular health conditions, including UM. Moreover, the protein content of EVs displays distinct glycosignatures [15]. Since specific changes in glycosylation are considered the major hallmark of cancer, the glycoproteome of EVs appears as another source of novel tumor biomarkers.

Considering potential role of EVs in the development and progression of UM, we chose to investigate the protein content and glycosylation pattern of ectosomes, a heterogeneous population of vesicles ranging from 0.1 to 1 μm in diameter. We performed LC-MS/MS-based proteomic analysis and used a panel of lectins to investigate specific glycosignatures of ectosomes released in vitro by a primary Mel202 UM cell line.

## 2. Results

### 2.1. Transmission Electron Microscopy (TEM) of Mel202-Derived Ectosomes

**Figure 1:**
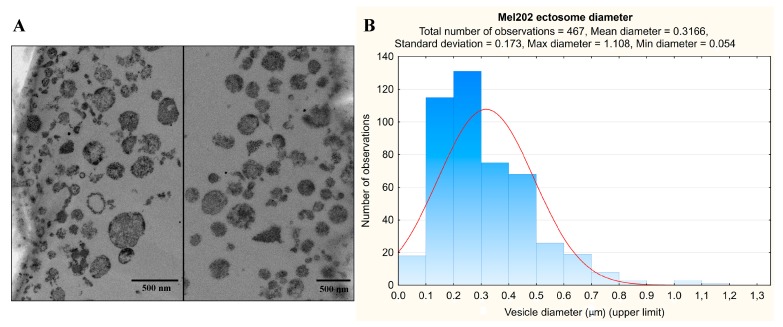
TEM analysis of Mel202-derived ectosomes. Samples were fixed with 2.5% glutaraldehyde, postfixed in 1% osmium tetroxide, dehydrated by passing them through a graded ethanol series, and embedded in epoxy resin. Ultrathin sections were contrasted using uranyl acetate and lead citrate. The sections were viewed on a JEOL JEM 2100HT TEM at 80 kV (**A**). Subsequently, diameters of *n* = 467 ectosomes were measured with the use of ImageJ software (**B**).

TEM analysis was done to validate the purity and effectiveness of ectosome isolation from the conditioned media via sequential centrifugation. Ultrathin sections of Mel202-derived ectosomes contained significant amounts of easily distinguishable extracellular vesicles, ranging from 54 to 1108 nm in diameter (Figure 1). The observed populations were rather heterogeneous and contaminated neither with intact cells nor with any cellular organelles. Among the 467 measured vesicles, only a few smaller than 100 nm and larger than 1 µm in diameter were found, indicating that the isolation protocol was properly performed.

### 2.2. Identified Proteins in Mel202-Derived Ectosomes and Their Functional Classification

A gel-free nano-LC-MS/MS proteomic approach was applied to profile ectosomal proteins secreted into the cell culture media using a primary Mel202 cell line. The submission of five datasets (Supplementary Data, Tables S1–S5), followed by redundancy removal, allowed us to generate a list of 2527 unique proteins (Supplementary Data, Table S6). A total of 949 proteins were identified by all five LC-MS/MS measurements (Supplementary Data, Table S7), suggesting their abundance in Mel202-derived ectosomes. The mean variability between technical replicates was assessed at 29.6%. Total variability between biological replicates was 44.6% and included the mean technical variability (29.6%) and 15% of actual biological variability (Supplementary Data, Table S8).

**Figure 2:**
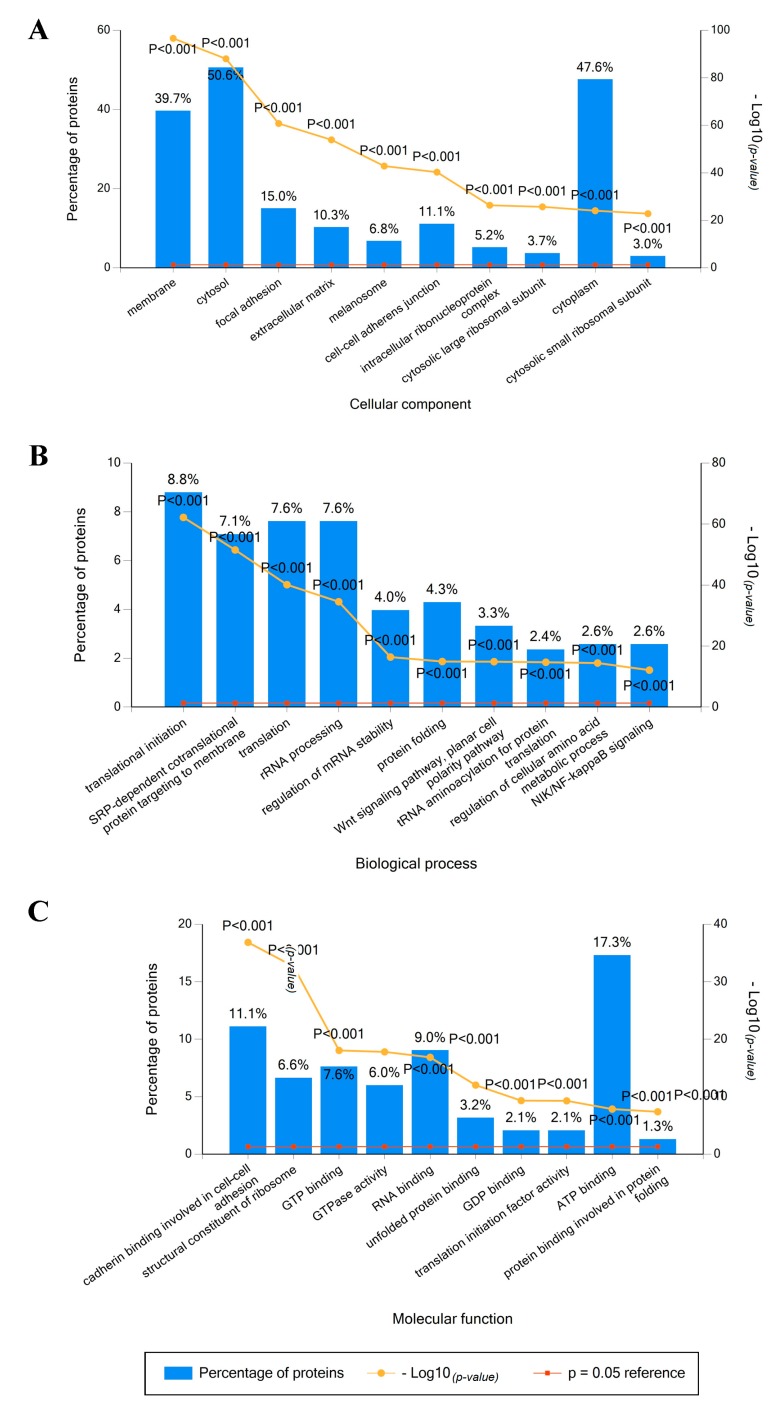
Gene ontology analysis of Mel202-derived ectosomal proteins using the UniProt protein database. Proteins were grouped according to the cellular compartments of their origin (**A**), involvement in different biological processes (**B**), and molecular functions (**C**). In each case, the ten most abundant groups (according to the percentage of assigned proteins) were presented on each graph. The *p*-value depicts the statistical significance of protein enrichment within the given categories.

**Figure 3:**
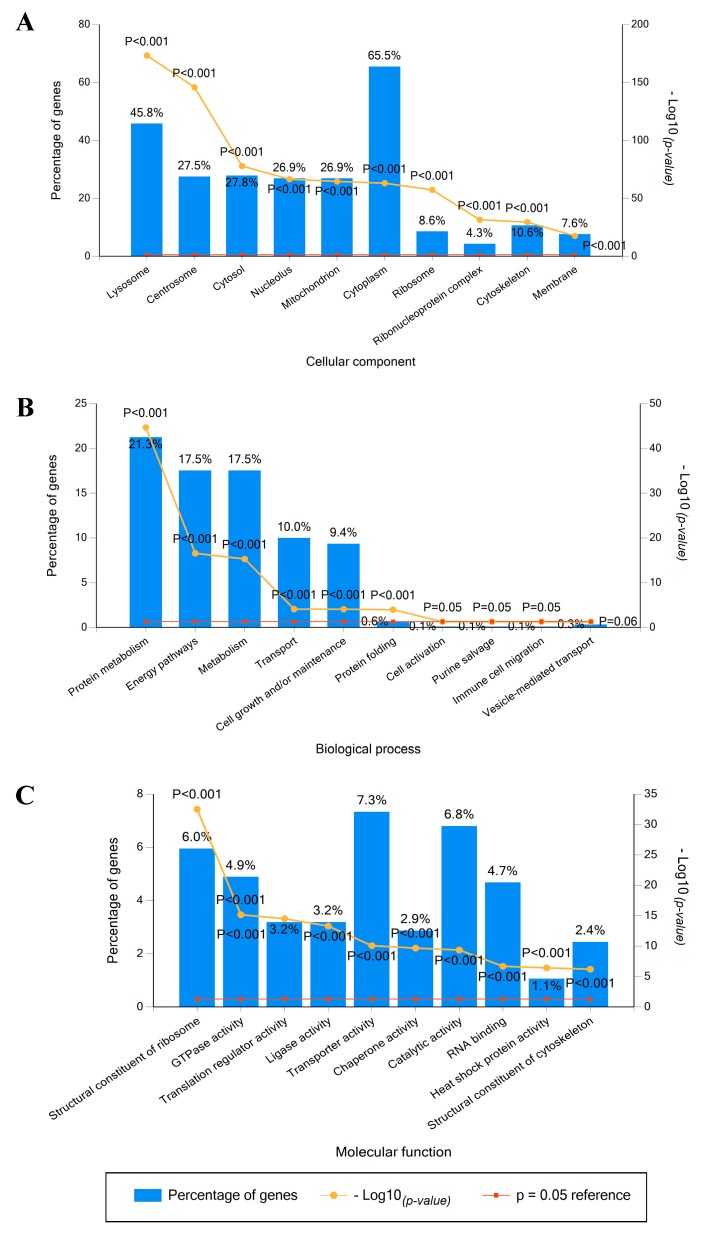
Gene ontology analysis of Mel202-derived ectosomal proteins using the FunRich gene database. Proteins were grouped according to the cellular compartments of their origin (**A**), involvement in different biological processes (**B**), and molecular functions (**C**). In each case, the ten most abundant groups (according to the percentage of assigned proteins) were presented on each graph. The *p*-value depicts the statistical significance of protein enrichment within the given categories.

Based on the data retrieved from the protein (UniProt) and gene (FunRich) databases, the 949 identified proteins were grouped according to the cellular compartments of their origin, molecular functions, and involvement in different biological processes. The obtained results varied due to different content of the bioinformatic tool but allowed for consistent conclusions. In both cases, the largest groups of Mel202-derived ectosomal proteins were associated with cytoplasmic/cytosolic (up to 65.5% of identified proteins) or membrane origin (up to 39.7%) (Figure 2A and Figure 3A). These findings reflect the mechanism of ectosome biogenesis that entails the accumulation of their components in particular domains within the membranes of origin. In support of the plasma membrane origin of ectosomes, many of the identified proteins were associated with cell–cell adhesion including adherens junctions (11.1%), focal adhesion (15%), or cadherin-binding activity (11.1%).

Regarding the involvement in different biological processes (Figure 2B and Figure 3B), the abundant group of 9.4% of proteins was related to a widely defined cell growth and maintenance. This dominance was further reflected in a more detailed classification, as a majority of proteins was matched to categories related to protein metabolism and energy pathways, as well as RNA processing and translation-related events. When classified by their precise molecular functions (Figure 2C and Figure 3C), a significant number of identified proteins had nucleic acid (mainly RNA) or nucleotide-binding (ATP, GTP) activity. In addition, Mel202-derived ectosomes were enriched in proteins displaying chaperone activity, mainly heat shock proteins (HSPs). This observation is in line with recent findings suggesting EVs as a form of cellular communication during cancer-associated stress conditions, with HSP70 being intensively exported [16].

### 2.3. Cancer-Associated Proteins in Mel202-Derived Ectosomes

The proteomic analysis of Mel202-derived ectosomes allowed for the identification of many interesting proteins that might influence the biological behavior of primary UM. The dbDEPC 3.0 database (designed to store and display proteins differentially expressed in cancer) was used to select cancer-related proteins and then classify them according to their role in tumor progression. Sixty-six cancer-related proteins (6.95% out of 949) comprised the proteins involved in cell proliferation/apoptosis, invasion, metastasis, altered metabolism, drug resistance, and chaperone activity (Table 1, detailed description in Supplementary Data, Table S9).

### 2.4. Glycosylation of Mel202-Derived Ectosomes and the Parental Cell Membranes

**Figure 4:**
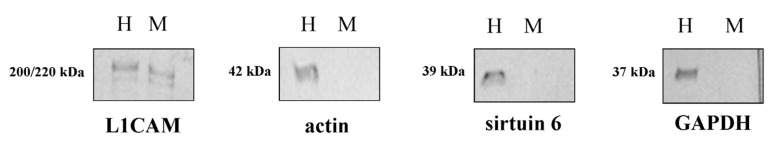
Assessment of the membrane fraction purity. On-blot immunodetection of proteins representative of each subcellular fraction was performed for the whole-cell extracts and the membrane fractions obtained from Mel202 cells. An antibody against L1CAM was used as a control for the membrane fraction, an antibody against actin for the cytoskeletal fraction, antibody against sirtuin-6 for the nuclear fraction, and an antibody against glyceraldehyde 3-phosphate dehydrogenase (GAPDH) for the cytosolic fraction.

The whole cell protein extract, the parental cell membrane protein fraction, and ectosomal proteins from Mel202 cells were analyzed using lectin blotting to investigate their glycosignatures. First, to assess the purity of the obtained cell membrane protein fraction, immunoblotting analysis was performed with the use of antibodies against proteins representative for each subcellular fraction (Figure 4, original blots in Supplementary Data, Table S10). As expected, L1CAM, a transmembrane cell adhesion molecule, was detected as a doublet with bands at 200 and 220 kDa in the whole cell extract and in the respective membrane fraction. The isolated membrane fraction did not contain any proteins specific for any other subcellular fractions; therefore, it was used for further analysis via lectin blotting.

**Figure 5:**
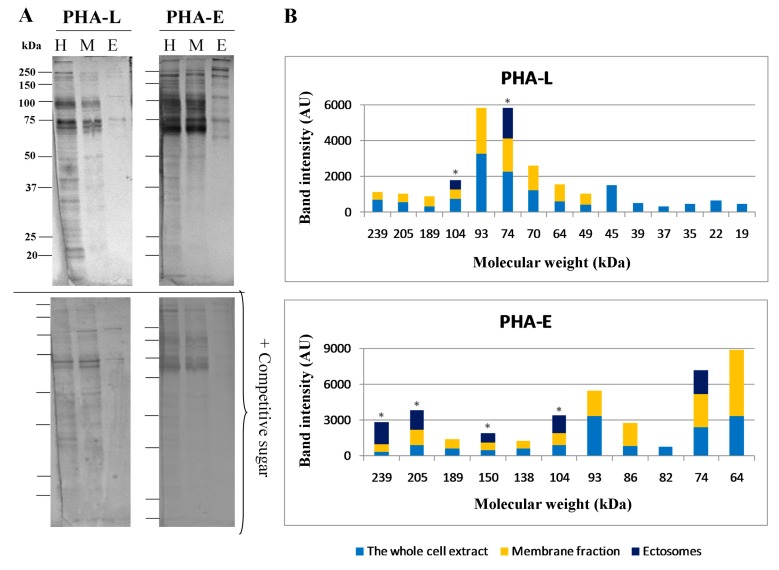

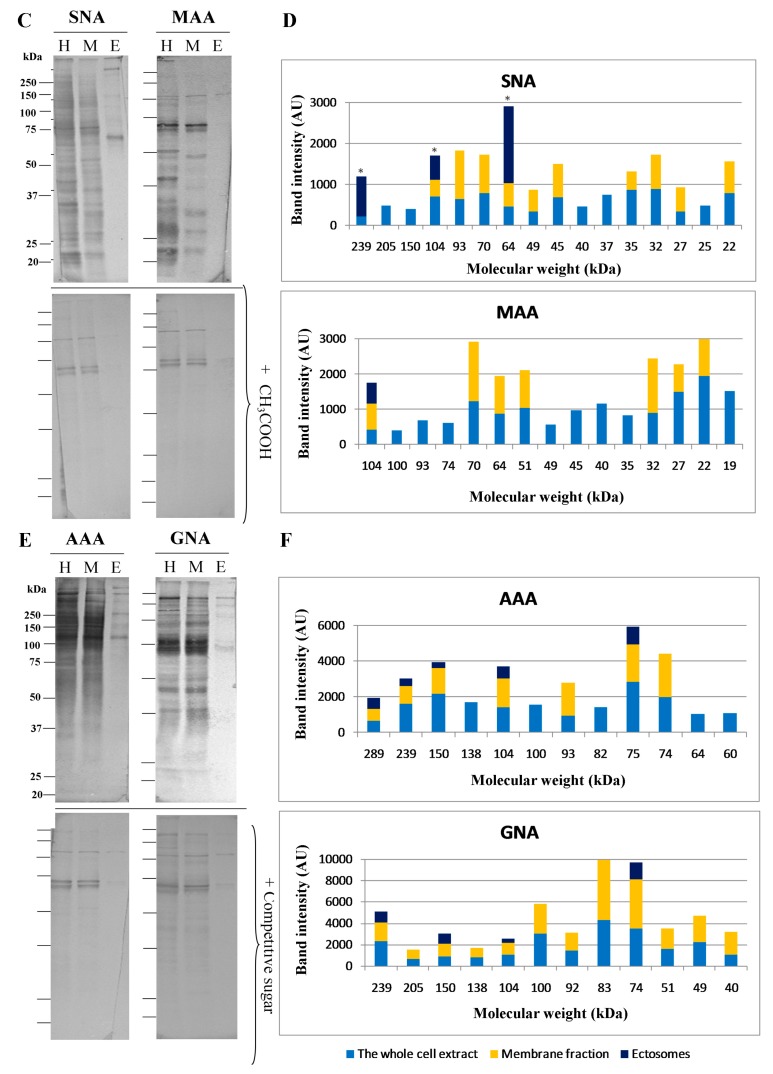
Comparative analysis of lectin-specific glycosignatures in Mel202-derived ectosomes. Reactions with biotinylated (**A**) PHA-L and PHA-E, (**C**) SNA and MAA, and (**E**) AAA and GNA, where lectins are shown in the upper panels and controls with blocking solution of competitive sugars or CH_3_COOH (as indicated in Supplementary Data 1) are shown in the lower panels. Seventy micrograms of protein were loaded per lane. Detection was performed via nitro-blue tetrazolium and 5-bromo-4-chloro-3′-indolyphosphate (NBT/BCIP) staining. H—the whole cell extract, M—membrane fraction, E—ectosomes. Densitometric analysis of (**B**) PHA-L- and PHA-E-, (**D**) SNA- and MAA-, and (**F**) AAA- and GNA-positive bands. The intensity of detected bands was expressed in arbitrary units (AU) as a peak area on a respective densitogram. Each bar represents the intensity of corresponding bands in the whole cell extract, membrane fraction, and ectosomes from Mel202 cells. * denotes bands that were enriched in ectosomes, relative to the membrane fraction. The values from control lectin blots with blocking solutions were subtracted beforehand.

The reaction with *Phaseolus vulgaris* leukoagglutinin (PHA-L) confirmed the presence of β1,6-branched tri- and/or tetra-antennary complex type N-glycans in the Mel202 whole-cell extract and the membrane fraction (Figure 5A, original blots in Supplementary Data, Table S10), and the number of PHA-L-positive bands was reduced in the membrane fraction. In Mel202-derived ectosomes, two PHA-L-positive bands were detected at 74 and 104 kDa, both of which showed a higher intensity in comparison to corresponding bands from the whole cell extract and the membrane fraction (Figure 5B). Subsequently, *Phaseolus vulgaris* erythroagglutinin (PHA-E) was used to detect proteins possessing bisecting GlcNAc in complex type N-glycans. For the whole cell extract and membrane fractions, a strong positive reaction was detected above ≈60 kDa and lectin binding was significantly decreased in the presence of the competitive sugar (Figure 5A). Moreover, PHA-E-positive glycoproteins were enriched in ectosomes relative to the whole-cell extract and the membrane fractions (Figure 5B). Taken together, the present results might suggest that proteins possessing β1,6-branched tri- and/or tetra-antennary complex type N-glycans and glycan structures with bisecting GlcNAc may be preferentially recruited into Mel202-derived ectosomes.

*Sambucus nigra* agglutinin (SNA) and *Maackia amurensis* agglutinin (MAA) were used to detect proteins carrying α2,6- and in α2,3-linked sialic acids, respectively (Figure 5C). The reaction with SNA revealed the enrichment of three bands at 64, 104, and 239 kDa in Mel202-derived ectosomes (Figure 5D). The reaction with MAA showed that the whole cell extract and the membrane fraction contained less protein bands possessing α2,3-linked sialic acids than protein bands possessing α2,6-linked sialic acids (reaction with SNA). In the ectosome sample only, one MAA-positive band was detected that did not display the enrichment in α2,3-linked sialic acids (Figure 5D).

A strong positive reaction with *Galanthus nivalis* agglutinin (GNA) confirmed the presence of proteins bearing high-mannose and/or hybrid type oligosaccharides in the whole-cell extract and the membrane fraction (Figure 5E). Mannose-rich glycoproteins were sorted into ectosomes but none of four bands (74, 104, 150, and 239 kDa) showed a higher intensity (Figure 5F).

The presence of proteins possessing fucose residues was revealed by the reaction with *Aleuria aurantia* agglutinin (AAA) (Figure 5E). In the whole cell extract and the membrane fraction, a strong reaction was observed with only three nonspecific bands, as revealed by the control with the competitive sugar. Regarding ectosomal proteins, five AAA-positive bands (at 75, 104, 150, 239, and 289 kDa) were detected; however, this was with a lower intensity when compared to corresponding bands in the whole-cell extracts and the membrane fractions (Figure 5F).

**Figure 6:**
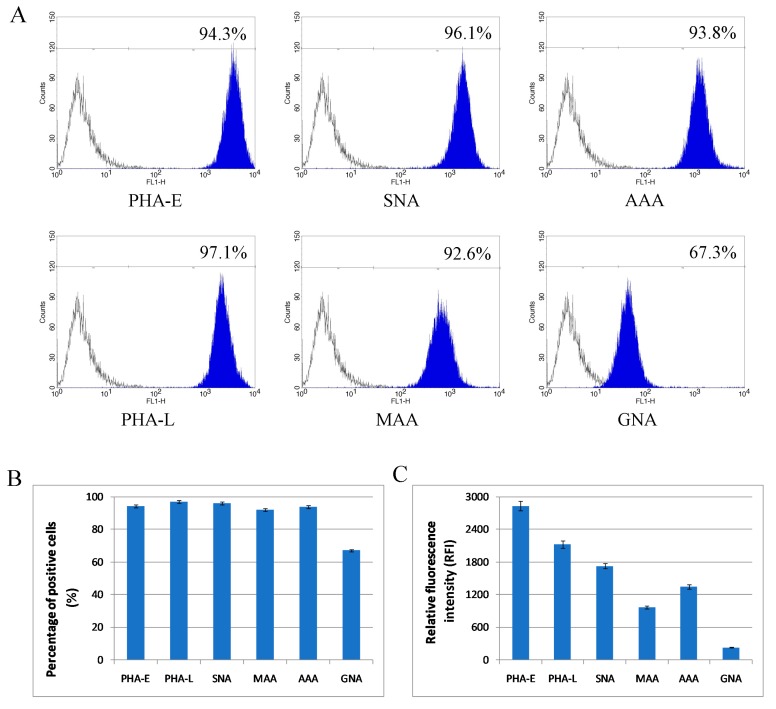
Flow cytometry analysis of surface glycosylation of Mel202 cells. (**A**) Histograms for MAA-, SNA-, GNA-, AAA-, PHA-E-, and PHA-L-positive cells. Colored areas represent staining with a panel of six lectins, while open histograms represent background fluorescence of the unspecific binding of extravidin-fluorescein isothiocyanate (FITC). The x-axis shows log fluorescence intensity and the y-axis shows the number of events. (**B**) Diagram showing the percentage of positive cells in each staining. (**C**) Diagram showing the relative fluorescence intensity (RFI) for each staining.

Additionally, flow cytometry was used to verify to what extent the revealed enrichment or depletion of specific glycoepitopes in Mel202-derived ectosomes in comparison to the parental cell membranes was related to the surface (plasma membrane-associated) glycoproteins. Reactions with the same panel of lectins revealed that >92% of Mel202 cells possessed α2,3- and α2,6-linked sialic acids, fucose residues, and bisecting GlcNAc in complex type N-glycans, as well as β1,6-branched tri- and/or tetra-antennary complex type N-glycans (Figure 6A,B). However, the number of GNA-positive cells was much smaller in comparison to staining with other lectins.

The measurement of relative fluorescence intensity (RFI) revealed a greater diversity related to the amount of given glycoepitopes on the cell surface (Figure 6C). Complex type N-glycans with bisecting GlcNAc (PHA-E staining), β1,6-branched N-glycans (PHA-L staining), and fucose residues (AAA staining) were the most abundantly present on the surface of Mel202 cells. Regarding sialic acid residues, SNA-related RFI acid was higher than MAA-related RFI, suggesting the dominance of α2,6-linked sialic acid on the Mel202 cell surface. Finally, the lowest RFI was measured for GNA-positive cells, suggesting that high-mannose and/or hybrid type oligosaccharides were mostly intracellular.

**Figure 7:**
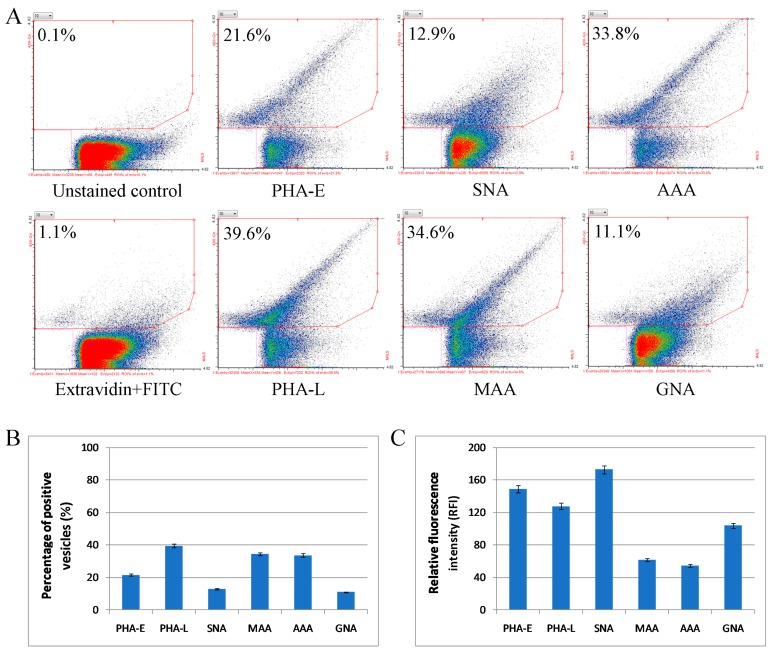
Flow cytometry analysis of surface glycosylation of Mel202-derived ectosomes. (**A**) Dot plots for MAA-, SNA-, GNA-, AAA-, PHA-E-, and PHA-L-positive ectosomes. Results for unstained control and control secondary extravidin-FITC staining are also provided. The x-axis shows values obtained using medium angle light scatter (MALS) and the y-axis shows log fluorescence intensity measured on 488Gn laser. (**B**) Diagram showing the percentage of positive ectosomes in each staining calculated via the subtraction of the value for the unspecific binding (secondary FITC-extravidin staining) from the raw value for each lectin staining. (**C**) Diagram showing the relative fluorescence intensity (RFI) for each staining.

Considering Mel202-derived ectosomes, the percentage of lectin-positive vesicles (<40% for each staining) was highly decreased in comparison to the analogous staining of Mel202 cells (Figure 7). The decrease was the most prominent in the case of complex type N-glycans with bisecting GlcNAc (PHA-E staining) (approx. 5-fold) and α2,6-linked sialic acids (SNA staining) (approx. 10-fold). SNA staining of Mel202-derived ectosomes was, however, characterized by the highest RFI alongside PHA-E and PHA-L staining. Opposite to Mel202 cells, where RFI of GNA staining was the lowest among the panel of lectins, RFI of GNA-positive ectosomes was higher than RFI for MAA and AAA staining.

## 3. Discussion

The present study was the premier attempt at characterizing protein and glycan content of ectosomes released in vitro by UM Mel202 cells. So far, the only studies on UM-derived EVs concerned the miRNA content of exosomes isolated from the vitreous humor of enucleated UM-affected eyes [17] or from liver circulation of patients with liver metastasis [18]. Proteomic profiling of the entire UM secretome from patients’ sera [3] and primary cell cultures [6,7] were also performed; however, in both studies, no particular EVs population was isolated. The most successful studies on UM proteome identified over 1600 [11] and 1800 [6] proteins in tissue samples or in media of primary cell cultures, respectively. Since our study analyzed only one population of released EVs, the presented method of proteomic analysis, in which 2527 unique proteins were identified, proved to be highly effective.

Our study showed that Mel202-derived ectosomes contained 13 cancer-related proteins that were associated with either cancer cell survival or death. Notably, two forms of annexin with antagonistic effects during cancer progression were identified: annexin A1, which inhibits antiapoptotic signaling pathways, and annexin A2, which promotes cancer cell proliferation [19].

Another 27 cancer-related proteins were associated with cancer invasion and metastasis. These proteins are directly involved in cell-cell and cell-matrix adhesion (e.g., galectin-1 and -3, integrin subunits β1 and αV, calreticulin, glycoprotein MUC18); matrix breakdown (cathepsin B and D); and cytoskeletal reorganization (cofilin-1, filamin-A, profilin-1, spectrin, vimentin). The presence of vimentin and the lack of keratin 8 and 18 confirms the primary phenotype of Mel202 cells because vimentin/keratin co-expression is linked with the high metastatic potential of UM [20,21]. Mel202-derived ectosomes also carried syntenin-1, a regulator of tumor invasion and angiogenesis, whose expression correlates with metastatic progression [22].

Mass spectrometry data for Mel202-derived ectosomes also revealed the presence of galectin-3 (Gal-3) and galectin-3-binding protein (LGALS3BP). Gal-3 has not been studied in UM; however, it was shown that the silencing of Gal-3 expression in cutaneous melanoma cells resulted in the loss of invasiveness, metastatic potential, and vasculogenic mimicry [23]. Gal-3 is also able to increase the motility of these cells by regulating matrix metalloproteinase (MMP)-1 expression [24]. Our study identified only one metalloproteinase, i.e., MMP-14, in Mel202-derived ectosomes. Nevertheless, UM cells has been shown to secrete MMP-2 and MMP-9, as well as CD147 (main inducer of MMPs activity), and their expression levels showed the correlation with UM metastatic potential and prognosis [25,26].

We also demonstrated that Mel202-derived ectosomes contained multiple proteins, including PI3K, which are associated with the Warburg effect, hyperactive glycolysis, and/or hypoxia-induced metabolic changes. Furthermore, Mel202-derived ectosomes were rich in proteins with chaperone activity, including proteins from the major heat shock protein (HSP) families: HSP60, HSP70, and HSP90. So far, a few studies concerning HSPs and UM have focused only on their diagnostic/prognostic values and will be discussed in the following section.

Finally, three multidrug resistance (MDR)-related proteins were identified in Mel202-derived ectosomes. MDR cancer cells have already been shown to overexpress different transporter proteins involved in the efflux of anticancer drugs including multidrug resistance-associated protein 1 (MRP1) [27]. MRP1 was present in Mel202-derived ectosomes and previous studies showed the association of higher MRP1 levels in biopsy specimens with the aggressiveness and spread of metastatic melanoma [28].

Much effort is now being directed at finding markers in primary tumors that could indicate their metastatic potential. Since protein content of EVs is dependent on the cells of origin, ectosomes should be considered as an alternative source of existing and/or novel UM biomarkers.

One of the first potential UM markers identified by proteomic studies was HSP27 [8]. Our studies failed to identify HSP27; however, several other HSPs were present, including HSP beta-1 that was recently described as differentially expressed in primary and metastatic UM tumors [11]. More recently, proteomic analysis of primary UM tumor samples from patients who developed and who did not develop metastatic disease within 7 years revealed 14 proteins that were either upregulated or underexpressed in samples from patients who developed metastasis [12]. Seven of these proteins (alpha enolase, triosephosphate isomerase, protein DJ-1, fatty acid-binding protein, eukaryotic translation initiation factor 2 subunit 1, 40S ribosomal protein SA, and tubulin beta chain) were identified in Mel202-derived ectosomes.

Furthermore, proteomic studies on primary and metastatic UM cell lines showed a higher expression of MUC18, HSP60β, and high mobility group protein 1 (HMG-1) in metastatic cell lines [7], and all these proteins were present in Mel202-derived ectosomes. Another novel biomarker identified in Mel202-derived ectosomes is DJ-1 protein. Elevated levels of DJ-1 have been associated with the malignant transformation of choroidal naevus, suggesting the importance of serum DJ-1 in monitoring patients at risk for ocular malignancies [29]. Mel202-derived ectosomes also carried cathepsin D and cofilin-1. The higher levels of cathepsin D were observed in UM patients’ serum in comparison to healthy subjects [3], while cofilin-1 was upregulated in metastatic UM cell lines [8].

The latest trend in UM proteomics analysis concerns the changes in protein content upon irradiation with lactate dehydrogenase B (LDHB) showing the highest fold-change in irradiated cells [10]. LDHB is involved in glycolysis and the increase in LDHB level may be related to a halt in the growth and progression of UM. LDHB was identified in Mel202-derived ectosomes, suggesting their role in transferring the molecular characteristics of UM cells that undergo cell-cycle suspension. Identification of other interacting proteins may provide alternative treatment strategies for primary UM and reduce the need for radiation therapy.

EVs display distinct glycosignatures, which could be another target in identifying novel cancer biomarkers. Studies on exosomes released by transformed T-cells [30], cutaneous melanoma [30,31], colon [30], and ovarian cancer cells [32,33] showed the enrichment or depletion of specific glycan epitopes in comparison to the parental cell membranes. Major observations included the enrichment in high-mannose and complex type N-linked glycans as well as α2,6-linked sialic acids and poly-N-acetyllactosamine epitopes. Also, our recent studies revealed the enrichment (mainly with fucose and complex type N-glycans with bisecting GlcNAc) of ectosomal proteins from four cutaneous melanoma cell lines [15].

Regarding glycan structures that promote protein incorporation into EVs, complex type N-glycans are the most widely studied. The treatment of OVMz cells with kifunensine, which prevents the processing of high-mannose to complex type N-glycans, decreased the levels of several N-linked glycoproteins in EVs but not the levels of non-glycosylated annexin-I [33]. Similarly, deoxymannojirimycin, an α-mannosidase I inhibitor, decreased the level of EWI-2 glycoprotein in Sk-Mel-5-derived exosomes, while its level on the cell surface was not affected. Sk-Mel-5-derived exosomes were also enriched in α2,6-linked sialic acids and in high-mannose type N-glycans; however, these observations have not been confirmed in ectosomes so far [31].

Our lectin-based studies revealed a distinct glycosylation pattern between Mel202-derived ectosomes and the parental cell membranes, and the enrichment of ectosomal proteins with complex type N-glycans with bisecting GlcNAc and α2,6-linked sialic acids. As each glycoprotein band detected in ectosomes was also present in the membrane fractions, it is likely that any intracellular glycoproteins were initially recruited into the plasma membrane before being sorted to ectosomes. Flow cytometry analysis showed that the percentage of lectin-stained Mel202-derived ectosomes was generally decreased in comparison to the analogous staining of the parental cell surface, and similar observations was recently made for cutaneous melanoma-derived ectosomes [15]. Because two types of flow cytometers were used for measurements and the analyzed objects have different size range, it is not possible to compare the obtained RFI values for Mel202 cells and Mel202-derived ectosomes. Nevertheless, RFI values for Mel202-derived ectosomes revealed the abundance of α2,6-linked sialic acids, complex type N-glycans with bisecting GlcNAc, high mannose type, and β1,6-branched N-glycans on their surface.

Glycans play a fundamental role during tumor cell dissociation and invasion, mainly by interfering with intercellular signaling and cell–cell adhesion. For instance, higher expression of β1,6-branched N-glycans interferes with UM cells migration and causes their weaker binding to fibronectin and vitronectin [34,35,36]. In the present study, β1,6-branched and complex type N-glycans with bisecting GlcNAc were detected in Mel202-derived ectosomes and several PHA-L- and PHA-E-positive bands showed a significant enrichment. The release of glycoproteins with bisecting GlcNAc via ectosomes might be a mechanism that allows cancer cells to retain their metastatic potential.

Changes in sialylation and fucosylation are also associated with cancer progression. The fucosylated and sialylated structures often form the sialylated Lewis determinants that participate in cell adhesion and the formation of metastases [37]. Our lectin-blotting results revealed the enrichment of several sialoglycoproteins with α2,6- but not with α2,3-linked sialic acids in Mel202-derived ectosomes. Despite the presence of AAA- and GNA-positive bands, neither fucose- nor mannose-containing glycans were enriched in Mel-202-derived ectosomes. In contrast, GNA-positive bands were detected in ectosomes derived from cutaneous melanoma cell lines with various degrees of malignancy [15]. The question whether the selective ectosomal transfer of glycans is determined by cancer type or disease stage remains to be addressed in further studies.

## 4. Materials and Methods 

### 4.1. Cell Lines and Cell Culture Conditions

Primary uveal melanoma Mel202 cell line was obtained from the ESTDAB Melanoma Cell Bank (Tübingen, Germany). Cells were maintained in RPMI 1640 medium with GlutaMAX-I (Thermo Fisher Scientific, Waltham, MA, USA), supplemented with 10% fetal bovine serum (FBS), (Thermo Fisher Scientific, Waltham, MA, USA) and antibiotics (100 units/mL penicillin and 100 μg/mL streptomycin, both from Sigma-Aldrich, St. Louis, MO, USA). Cells were grown in monolayers in 5% CO_2_ atmosphere at 37 °C in a humidified incubator and passage after reaching 80% of confluence.

### 4.2. Isolation of Ectosomes and Verification of the Purity of the Sample

Sub-confluent cells were cultured for 24 h in serum-free RPMI 1640 medium supplemented with GlutaMAX-I and antibiotics. Conditioned media (approximately 1000 mL) were collected and subjected to sequential centrifugation steps at 400× *g*, 4000× *g*, 7000× *g*, and 18,000× *g*, as previously described in Surman et al. [15]. The purity of the obtained sample was verified by transmission electron microscopy (TEM) as previously described in Surman et al. [15].

### 4.3. Mass Spectrometry Analysis

#### 4.3.1. Ectosome Lysis

The pellets were washed three times with phosphate-buffered saline (PBS) and suspended in 50 µL of lysis buffer (100 mM Tris/HCl, pH 7.6, 1% *w*/*v* sodium dodecyl sulphate (SDS)). Lysates were sonicated with Bioruptor UCD-200 (Diagenode, Liège, Belgium) for 20 min at high intensity (320 W, 30 s/30 s on/off). Next, the samples were denatured at 95 °C under strong agitation for 5 min and centrifuged at 20,000× *g* for 10 min at 20 °C. Supernatants were collected and proteins were precipitated (one volume of trichloroacetic acid (TCA) was added to four volumes of the sample). After overnight incubation at −20 °C, the samples were spun at 10,000× *g* for 15 min at 10 °C and washed two times with ice-cold acetone. The pellets were resuspended in 100 µL of 10 mM HEPES (4-(2-hydroxyethyl)-1-piperazineethanesulfonic acid) pH 8.5.

#### 4.3.2. Sample Preparation for Mass Spectrometric Analysis

The samples were prepared using paramagnetic bead technology based on the Single-Pot Solid-Phase-Enhanced Sample Preparation (SP3) [38]. Two types of SpeedBeads™ mixed at a 1:1 ratio were used: GE45152105050250 and GE65152105050250 (Sigma-Aldrich). The proteins were reduced with dithiothreitol, alkylated with iodoacetamide, and digested with Trypsin/Lys-C Mix (Promega, Mannheim, Germany).

#### 4.3.3. Liquid Chromatography and Tandem Mass Spectrometry (LC-MS/MS)

Peptides were analyzed using UltiMate 3000 RSLCnano System coupled with Q-Exactive mass spectrometer (Thermo Fisher Scientific) with DPV-550 Digital PicoView nanospray source (New Objective, Wobum, MA, USA.). Samples were loaded onto a trap column (Acclaim PepMap 100 C18, 75 μm × 20 mm, 3 μm particle, 100 Å pore size, Thermo Fisher Scientific) in 2% acetonitrile with 0.05% trifluoroacetic acid (TFA) at a flow rate of 5 μL/min and further resolved on an analytical column (Acclaim PepMap RSLC C18, 75 µm × 500 mm, 2 µm particle, 100 Å pore size, Thermo Fisher Scientific) with a 90 min gradient from 2% to 40% acetonitrile in 0.05% formic acid at a flow rate of 200 nL/min. The Q-Exactive was operated in a data-dependent mode using a top-eight method. Full-scan MS spectra were acquired with a resolution of 70,000 at *m*/*z* 200 with an automatic gain control (AGC target) of 1 × 10^6^. The MS/MS spectra were acquired with a resolution of 35,000 at *m*/*z* 200 with AGC target of 3 × 10^6^. The maximum ion accumulation times for the full MS and the MS/MS scans were 120 ms and 110 ms, respectively. Peptides were dynamically excluded from fragmentation within 30 s. High-performance liquid chromatography (HPLC) solvents were of LC/MS grade and were purchased from JT Baker (Phillipsburg, NJ, USA).

#### 4.3.4. Analysis of the Proteomic Data

The raw data files were processed by the Proteome Discoverer platform (v.1.4, Thermo Fisher Scientific) and searched against the SwissProt database with Homo sapiens taxonomy restriction (release June 2017, 20,206 sequences) using the locally installed MASCOT search engine (v.2.5.1, Matrix Science). The following parameters were applied: fixed modification—cysteine carbamidomethylation; variable modifications—methionine oxidation and protein N-terminal acetylation; peptide mass tolerance—10 ppm; fragment mass tolerance—20 mmu. Only tryptic peptides with up to one missed cleavage were considered. Target Decoy PSM Validator was applied with the maximum false discovery rate (FDR) for peptides set to 0.01. The mass spectrometry proteomics data have been deposited to the ProteomeXchange Consortium via the PRIDE [39] partner repository with the dataset identifier PXD012003.

#### 4.3.5. Bioinformatic Analysis

Final protein list was created from data obtained after five LC-MS/MS measurements and included proteins that were identified by all biological and technical repetitions. The gene ontology analysis, with regard to the cellular compartment of origin, involvement in different biological processes and molecular function, was performed with the use of FunRich 2.0 software [40,41] with protein (UniProt, saved on July 23, 2018) and gene (FunRich, saved on July 23, 2018) databases as a reference. For each analysis, ten categories with the highest *p*-value (statistical significance of protein enrichment within the given categories) were presented on graphs. dbDEPC 3.0 (database of differentially expressed proteins in cancer, saved on 23 July 2018) was used to select cancer-related proteins identified in Mel202-derived ectosomes. Proteins that were differentially expressed in at least three different types of cancer (based on the referential MS data from dbDEPC 3.0) were considered cancer-related.

### 4.4. Lectin-Based Analysis of Mel202 Cell Extracts, Cell Membranes, and Ectosomes

The cell extract preparation, isolation of the membrane fraction, and assessments of its purity as well as lectin blotting and flow cytometry with the use of biotinylated *Phaseolus vulgaris* leukoagglutinin (PHA-L), *Phaseolus vulgaris* erythroagglutinin (PHA-E), *Sambucus nigra* agglutinin (SNA), *Maackia amurensis* agglutinin (MAA), *Aleuria aurantia* agglutinin (AAA), and *Galanthus nivalis* agglutinin (GNA) (all from Vector Laboratories Inc., Burlingame, CA, USA) were described previously in Surman et al. [15]. Sugar specificity and blocking solutions for the used panel of lectins as well as original blots are included in Supplementary Data, Table S10.

### 4.5. Statistical Analysis

Statistica 12 (StatSoft^®^) tools were used for all data analyses. The results are expressed as mean ± standard deviation of three independent experiments. The significant enrichment/depletion of glycan structures based on band intensities were computed using one-way ANOVA and post hoc Tukey’s test, and *p*-values < 0.05 were considered statistically significant.

## 5. Conclusions

Our work is the premier attempt on characterizing protein content and glycosylation of ectosomes released by primary UM Mel202 cells. Using nano-LC-MS/MS, we identified 2527 unique proteins, many of which were cancer-related and already implicated in different biological events associated with UM development and progression. Lectin-based studies allowed us to demonstrate a distinct glycosylation pattern between Mel202-derived ectosomes and the parental cell membranes.

Vesiculation is considered to be a very dynamic process. EV protein content along with its glycosylation are highly dependent on the current state of the parental cells, which makes it difficult to unequivocally indicate any conserved proteins and/or glycan structures within EVs. Nevertheless, such susceptibility to different physiological and pathological changes makes the EV glycoproteome a promising source of potential biomarkers. Therefore, exploring whether UM-related proteins and glycans are enriched in EVs isolated from different body fluids should become a subject of future in-depth studies.

## Figures and Tables

**Table 1:** Functional classification of 66 cancer-related proteins from Mel202-derived ectosomes. Proteins were selected with the use of the dbDEPC 3.0 database.

Cell Proliferation/Apoptosis	Cell Invasion and Metastasis	Cancer Cell Metabolism	Heat Shock Proteins and Chaperones	Drug Resistance	Others
Annexin A1 Annexin A2 Chloride intracellular channel protein 1 Heterogeneous nuclear ribonucleoprotein K Heterogeneous nuclear ribonucleoproteins C1/C2 Peptidyl-prolyl cis-trans isomerase A Peroxiredoxin-1 Peroxiredoxin-2 Peroxiredoxin-4 Peroxiredoxin-6 Serine/threonine-protein phosphatase 2A 65 kDa regulatory subunit A alpha isoform Stress-induced-phosphoprotein 1 T-complex protein 1 subunit beta	14-3-3 protein zeta/delta Calreticulin Calumenin Cathepsin B Cathepsin D CD44 antigen Cell surface glycoprotein MUC18 Cofilin-1 Filamin-A Galectin-1 Galectin-3 Galectin-3-binding protein Histone H4 Integrin alpha-V Integrin beta-1 Keratin, type II cytoskeletal 5 Nucleoside diphosphate kinase A Phosphatidylethanolamine-binding protein 1 Profilin-1 Ras GTPase-activating protein-binding protein 1 Rho GDP-dissociation inhibitor 1 Serpin H1 Syntenin-1 Spectrin beta chain Superoxide dismutase [Cu-Zn] Vimentin WD repeat-containing protein 1	Apolipoprotein E Fatty acid synthase Fructose-bisphosphate aldolase A Glyceraldehyde-3-phosphate dehydrogenase Isocitrate dehydrogenase (NADP) cytoplasmic Lactoylglutathione lyase L-lactate dehydrogenase A chain Nicotinamide phosphoribosyltransferase Phosphoglycerate kinase 1 Pyruvate kinase isozymes M1/M2	60 kDa heat shock protein, mitochondrial precursor Alpha-crystallin B chain Endoplasmin Heat shock 70 kDa protein 1A/1B Heat shock 70 kDa protein 4 Heat shock protein beta-1 Heat shock protein HSP 90-beta	Glutathione S-transferase omega-1 Multidrug resistance-associated protein 1 Proliferating cell nuclear antigen	Elongation factor 1-beta Epoxide hydrolase 1 Glutathione S-transferase P Heterogeneous nuclear ribonucleoproteins A2/B1 Nucleophosmin Transforming growth factor-beta-induced protein ig-h3

## Supplementary Materials

Supplementary materials can be found at https://www.mdpi.com/1422-0067/20/15/3789/s1. LC-MS/MS data are available via ProteomeXchange with identifier PXD012003.

## Abbreviations

AAA	*Aleuria aurantia* agglutinin
BCIP	5-bromo-4-chloro-3-indolyl-phosphate
EV	extracellular vesicles
Gal-3	galectin-3
GAPDH	glyceraldehyde 3-phosphate dehydrogenase
GNA	*Galanthus nivalis* agglutinin
HSP	heat shock protein
L1CAM	L1 cell adhesion molecule
LC-MS/MS	liquid chromatography coupled with mass spectrometry
LGALS3BP	galectin-3-binding protein
MAA	*Maackia amurensis* agglutinin
MDR	multidrug resistance
MMP	matrix metalloproteinase
NBT	4-nitro blue tetrazolium chloride
PHA-E	*Phaseolus vulgaris* erythroagglutinin
PHA-L	*Phaseolus vulgaris* leukoagglutinin
SDS-PAGE	sodium dodecyl sulfate polyacrylamide gel electrophoresis
SNA	*Sambucus nigra* agglutinin
TEM	transmission electron microscopy
UM	uveal melanoma
